# Differences in Medicaid Enrollment and Spending Before and During the COVID-19 Pandemic

**DOI:** 10.1001/jamanetworkopen.2025.16569

**Published:** 2025-06-18

**Authors:** Nianyi Hong, Noelia Duchovny, Ru Ding

**Affiliations:** 1 Congressional Budget Office, Washington, DC

## Abstract

**Importance:**

The COVID-19 pandemic and associated continuous eligibility policy were associated with lasting changes to Medicaid coverage and spending. These changes for both adults and children should be assessed to better inform policymakers of potential consequences over the long run.

**Objective:**

To compare enrollment and spending patterns for adults and children over the course of 18 months before and during the pandemic.

**Design, Setting, and Participants:**

This cohort study used Transformed Medicaid Statistical Information System administrative data to compare cohorts of Medicaid enrollees beginning in February 2018 compared with cohorts beginning in February 2020. Participants included adults and children enrolled in Medicaid in 25 states and the District of Columbia with reliable enrollment and spending data. The data were analyzed between August 31, 2023, and December 9, 2024.

**Exposures:**

The COVID-19 pandemic and Medicaid continuous eligibility policy.

**Main Outcomes and Measures:**

The primary outcomes were Medicaid enrollment and mean monthly Medicaid spending for cohorts of adults and children. Trends in enrollment and spending over an 18-month period were compared separately for adults and children. A decomposition was used to quantify the contribution of 2 factors—the share of people with any spending and mean spending among enrollees who had spending—to the observed spending differences across the 2 cohorts.

**Results:**

In month 1 of each cohort, the samples consisted of 12 352 041 adults (7 525 313 female [60.9%]) in the 2018 cohort, 11 998 538 adults (7 370 318 female [61.4%]) in the 2020 cohort, 14 917 138 children (7 367 579 female [49.4%]) in the 2018 cohort, and 14 585 026 children (7 205 186 female [49.4%]) in the 2020 cohort. The mean (SD) age of the 2018 and 2020 adult cohorts was 37.4 (12.4) years at month 1, and the mean (SD) ages of the 2018 and 2020 child cohorts were 8.8 (5.2) years and 9.0 (5.2) years at month 1, respectively. The pandemic and continuous eligibility policy increased the share of adults still enrolled in Medicaid after 18 months by 28 percentage points among adults and by 19 percentage points among children. The pandemic and policy were also associated with lower mean spending compared with a prepandemic cohort. This gap in spending was largest during the first few months of the pandemic with substantial lockdown policies (22 percentage points lower in adults and 36 percentage points lower in children). Finally, additional enrollees in month 18 of the 2020 cohort had spending that was 44% to 58% lower than that of existing enrollees. Although spending rebounded slightly from start of the pandemic, spending after 18 months still lagged below prepandemic levels.

**Conclusions and Relevance:**

In this cohort study, the continuous eligibility policy appeared to be effective at keeping individuals enrolled in Medicaid and additional enrollees had relatively lower spending. Incomplete recovery of spending suggested that crucial care may have been deferred.

## Key Points

**Question:**

How did patterns of enrollment and spending among a cohort of Medicaid enrollees change during the COVID-19 pandemic compared with an earlier cohort?

**Findings:**

In this cohort study with more than 12 million adults and 14 million children, the pandemic and its associated Medicaid continuous eligibility policy were associated with an increase in the share of people still enrolled in Medicaid after 18 months and lower mean spending. Over time, spending partially rebounded, but still lagged below prepandemic levels.

**Meaning:**

These findings suggest that the continuous eligibility policy kept people enrolled longer and enrollees had relatively lower spending, but crucial care may have been deferred.

## Teaser

This cohort study examines Medicaid enrollment and spending patterns for adults and children for 18 months before and during the COVID-19 pandemic.

## Introduction

At peak enrollment of Medicaid and the Children’s Health Insurance Program in March 2023, 94 million people were enrolled, an increase of 22 million since the start of the COVID-19 pandemic.^1^ Changes in economic conditions, new state Medicaid expansions, and the continuous eligibility provision in the Families First Coronavirus Response Act—through which states received an increase in the Federal Medical Assistance Percentage upon agreeing to keep all Medicaid recipients enrolled through the end of the public health emergency (PHE)—contributed to this increased enrollment. Recent studies^2,3,4,5,6,7,8^ indicate that most enrollment growth during this period could be attributed to decreases in disenrollment rather than increases in new enrollment.

Although the continuous eligibility provision and the pandemic increased the duration of Medicaid enrollment, less is known about their effects on mean Medicaid spending per enrollee, including both fee-for-service and managed care payments, as well specific effects among adults and children. (Related studies have examined the outcome of pandemic funding on state expenditures.^4,5^) Any changes in mean Medicaid spending would depend on factors including changes in the composition of enrollees, awareness of continued Medicaid coverage, and availability of other sources of insurance coverage.^2,3,7^ Mean spending could have decreased if people who stayed enrolled longer were healthier, whereas if people were sicker, the opposite would have occurred. Increased concurrent insurance coverage as people returned to work (leading Medicaid to be the payer-of-last-resort) and lack of awareness of Medicaid among people continuously enrolled could have also resulted in lower mean spending.^8,9^

Changes in mean spending during the pandemic were also affected by closures and lockdowns, which reduced spending for some enrollees, and direct health outcomes of the pandemic, which increased spending among enrollees who got sick.^10^ Such outcomes likely diminished over time as governments, businesses, and individuals took fewer actions addressing the pandemic.

In this study, we compared trends in enrollment and mean spending over an 18-month period for 2 cohorts of Medicaid enrollees—one before the pandemic and another during the pandemic. In addition, we calculated the extent to which changes in the share of enrollees with any spending and mean spending among enrollees who had any spending contributed to changes in mean spending and compared cumulative total spending for each cohort.

## Methods

This cohort study was reviewed by the Centers for Medicare & Medicaid Services (CMS) and was conducted consistent with the statutory authorities that govern access of the Congressional Budget Office (CBO) to CMS data, including section 201(d) of the Congressional Budget Act, 2 U.S.C. 601(d). In accordance with these authorities, CBO provides human participants the same level of confidentiality as the law requires of CMS, although CBO has not directly subjected itself to the Common Rule owing to the nature of its work and status as an office of the Congress. Thus, informed consent and institutional review board approval were not required. This study followed Strengthening the Reporting of Observational Studies in Epidemiology (STROBE) reporting guidelines.

Using administrative data on enrollment and claims, we monitored 2 cohorts of Medicaid enrollees: children (up to age 19 years) and adults who were either previously eligible for Medicaid or made newly eligible by the Patient Protection and Affordable Care Act. Elderly Medicaid beneficiaries and those with blindness and disability were not included because we expect those enrollees were less affected by the continuous eligibility provision and had less churn in Medicaid enrollment before those provisions. The first cohort consisted of enrollees as of February 2018, and the second consisted of enrollees as of February 2020. For both cohorts, we tracked changes in Medicaid enrollment and spending over an 18-month period, separately for adults and children. Specifically, 2 main outcomes of interest were the share of each cohort still enrolled in Medicaid in each month and mean spending per person per month conditional on being enrolled.

To further understand the changes in monthly spending, we calculated the contribution from changes in the number of enrollees with any spending and changes in mean spending among enrollees with any spending. See eAppendixes 1 to 4 in Supplement 1 for more details about the methods.

### Data and Analytical Sample

Our data were derived from the 2018 to 2021 Transformed Medicaid Statistical Information System Analytic Files Research Identifiable Files, which include detailed information on enrollment, Medicaid spending, and demographics, including race and ethnicity, for all states and the District of Columbia (we used release 2 of the 2018 data and release 1 of the 2019 to 2021 data). Our analytic samples included enrollees in 25 states and the District of Columbia with reliable data and without changes in eligibility criteria (see eFigure 1 and eTables 1 and 2 in Supplement 1 for more information). Data on race and ethnicity are included in this study to check that cohorts are similar from a demographic perspective.

The prepandemic cohort included Medicaid enrollees as of February 2018 (before the pandemic), and the pandemic cohort included Medicaid enrollees as of February 2020 (during the pandemic). By focusing on a sample enrolled in Medicaid before substantial employment loss in March 2020, we chose individuals who should be demographically like the prepandemic cohort. Enrollees in both cohorts were followed-up for 18 months. Individuals in our sample who churned off Medicaid were not added back to the sample if they re-enrolled (see eAppendix 2 and eTable 3 in Supplement 1 for more details).

### Outcomes

The key variables in our analyses were monthly enrollment and mean monthly spending. Because enrollees can receive services under fee-for-service (where the state pays practitioners directly for each covered service received by an enrollee) or under managed care (where the state pays a premium to a managed care plan for each person enrolled in the plan and the plan pays practitioners), we defined spending as the sum of fee-for-service payments and managed care payments to practitioners. Although payments to practitioners in managed care are not generally available to researchers, we had access to these data fields through our data user agreement. This definition captures changes in spending that may not be fully captured by changes in premiums because premium changes likely lag changes in spending.

To compare changes in outcomes between the cohorts, we normalized monthly enrollment and spending so that the first month’s enrollment and spending in each cohort were indexed to 100% and any changes in outcomes for months 2 to 18 were compared with month 1. This normalization facilitated the examination of attrition in enrollment over time and controlled for factors such as inflation and macroeconomic changes that may create level differences between first month mean spending between the 2 cohorts. Furthermore, before indexing spending in the 2020 cohort, we adjusted the spending in February 2020 to account for differences in number of days in February 2020 vs February 2018.

Other variables used in the analyses included demographic characteristics and an indicator for secondary insurance coverage. That indicator is based on the third-party liability insurance coverage variable in the Transformed Medicaid Statistical Information System Analytic Files eligibility file, is a monthly variable available for all enrollees (regardless of whether they submitted a claim), and is populated if an individual has other sources of insurance in addition to Medicaid, making Medicaid the payer-of-last-resort. (A separate variable, not used in the analyses, indicates whether the enrollee has Medicare coverage in addition to Medicaid.)

### Statistical Analysis

The data were analyzed between August 31, 2023, and December 9, 2024. We examined trends in monthly enrollment and mean spending for the 2018 and 2020 cohorts for a period of 18 months, separately for children and adults. Whether an enrollee was categorized as a child or adult was based on their age in the first month of the cohort; therefore, a child who turned 19 years old during the study period stayed in the child cohort and did not switch into the adult group.

We also used a decomposition to quantify the contribution of 2 factors—the share of people with any spending and mean spending among enrollees who had spending—to the observed spending differences across the 2 cohorts. This analysis was motivated by evidence suggesting that a greater number of people during the PHE were unable to access services especially at the beginning of the pandemic, were unaware of their Medicaid coverage, or had multiple sources of insurance coverage, all of which could have affected spending.^8,11^

Finally, we estimated how mean spending among the additional people enrolled in month 18 of the 2020 cohort compared with spending among enrollees in month 18 of the 2018 cohort. This calculation divided the share of people enrolled in month 18 of 2020 into the share that would have been enrolled in the absence of the pandemic and continuous eligibility policy as proxied by the 2018 shares and the additional share enrolled in month 18 of 2020. In addition, we assumed that the first group—people enrolled in month 18 of 2020 who would have been enrolled in the absence of the pandemic and continuous eligibility policy—had the same patterns of spending as the 2018 cohort. More details are included in eFigure 2 and eAppendix 3 in Supplement 1. Data were analyzed using SAS statistical software version 9.4 (SAS Institute).

## Results

The Table shows that the prepandemic and pandemic cohorts of adults and children were similar in terms of age, sex, race, and ethnicity. The sample consisted of 12 352 041 adults (7 525 313 female [60.9%]) in month 1 of the 2018 cohort, 11 998 538 adults (7 370 318 female [61.4%]) in month 1 of the 2020 cohort, 14 917 138 children (7 367 579 female [49.4%]) in month 1 of the 2018 cohort, and 14 585 026 children (7 205 186 female [49.4%]) in month 1 of the 2020 cohort. The mean (SD) age of the 2018 and 2020 adult cohorts was 37.4 (12.4) years at month 1, and the mean (SD) ages of the 2018 and 2020 child cohorts were 8.8 (5.2) years and 9.0 (5.2) years at month 1, respectively (Table).In the 2018 adult cohort, 803 700 participants (6.5%) were Asian, 1 874 633 participants (15.2%) were Black, 3 010 966 participants (24.4%) were Hispanic, 4 445 842 participants (36.0%) were White, 364 191 participants (3.0%) were other race (ie, any minoritized racial and ethnic group other than Asian, Black, or Hispanic), and 1 852 709 participants (15.0%) were missing data on race and ethnicity in month 1. In the 2020 adult cohort, 744 587 participants (6.2%) were Asian, 1 892 280 participants (15.8%) were Black, 2 981 588 participants (24.9%) were Hispanic, 4 429 263 participants (36.9%) were White, 381 420 participants (3.2%) were other race, and 1 569 400 participants (13.1%) were missing data on race and ethnicity in month 1. In the 2018 children’s cohort, 515 886 participants (3.5%) were Asian, 2 326 291 participants (15.6%) were Black, 4 915 207 participants (33.0%) were Hispanic, 3 993 395 participants (26.8%) were White, 374 673 participants (2.5%) were other race, and 2 791 686 participants (18.7%) were missing data on race and ethnicity in month 1. In the 2020 children’s cohort, 458 318 participants (3.1%) were Asian, 2 372 448 participants (16.3%) were Black, 4 788 473 participants (32.8%) were Hispanic, 3 846 266 participants (26.4%) were White, 404 854 participants (2.8%) were other race, and 2 714 667 participants (18.6%) were missing data on race and ethnicity in month 1. These characteristics changed minimally over the 18-month follow-up period, less so in the 2020 cohorts as almost all people stayed enrolled throughout the follow-up period.

**Table.** Characteristics of Prepandemic (2018) and Pandemic (2020) Cohorts^a^

Characteristic	Adults, No. (%)				Children, No. (%)			
	2018 Cohort		2020 Cohort		2018 Cohort		2020 Cohort	
	Month 1 (n = 12 352 041)	Month 18 (n = 7 942 092)	Month 1 (n = 11 998 538)	Month 18 (n = 11 065 744)	Month 1 (n = 14 917 138)	Month 18 (n = 11 259 064)	Month 1 (n = 14 585 026)	Month 18 (n = 13 767 655)
Age, mean (SD), y	37.4 (12.4)	39.9 (12.5)	37.4 (12.4)	39.1 (12.4)	8.8 (5.2)	10.3 (5.1)	9.0 (5.2)	10.5 (5.2)
Sex^b^								
Female	7 525 313 (60.9)	4 928 651 (62.1)	7 370 318 (61.4)	6 811 843 (61.6)	7 367 579 (49.4)	5 567 455 (49.5)	7 205 186 (49.4)	6 802 770 (49.4)
Male	4 826 728 (39.1)	3 013 441 (38.0)	4 628 220 (38.6)	4 253 901 (38.5)	7 549 559 (50.6)	5 691 609 (50.6)	7 379 840 (50.6)	6 964 885 (50.6)
Race and ethnicity^b^								
Asian	803 700 (6.5)	569 510 (7.2)	744 587 (6.2)	688 258 (6.2)	515 886 (3.5)	399 843 (3.6)	458 318 (3.1)	433 394 (3.2)
Black	1 874 633 (15.2)	1 186 893 (14.9)	1 892 280 (15.8)	1 762 002 (15.9)	2 326 291 (15.6)	1 771 653 (15.7)	2 372 448 (16.3)	2 240 180 (16.3)
Hispanic	3 010 966 (24.4)	1 995 496 (25.1)	2 981 588 (24.9)	2 756 016 (24.9)	4 915 207 (33.0)	3 713 991 (33.0)	4 788 473 (32.8)	4 549 820 (33.1)
White	4 445 842 (36.0)	2 793 558 (35.2)	4 429 263 (36.9)	4 086 003 (36.9)	3 993 395 (26.8)	2 982 121 (26.5)	3 846 266 (26.4)	3 623 617 (26.3)
Other^c^	364 191 (3.0)	246 745 (3.1)	381 420 (3.2)	355 784 (3.2)	374 673 (2.5)	292 915 (2.6)	404 854 (2.8)	382 756 (2.8)
Missing race	1 852 709 (15.0)	1 149 890 (14.5)	1 569 400 (13.1)	1 417 681 (12.8)	2 791 686 (18.7)	2 098 541 (18.6)	2 714 667 (18.6)	2 537 888 (18.4)
Has secondary coverage	1 334 475 (10.8)	780 772 (9.8)	1 486 552 (12.4)	1 585 946 (14.3)	1 436 390 (9.6)	1 037 187 (9.2)	1 435 449 (9.8)	1 569 107 (11.4)

^a^ Data are authors’ analysis of Transformed Medicaid Statistical Information System Analytic Files claims data, 2018 to 2021.

^b^ Estimates are rounded to the nearest tenth and might not add up to 100 because of rounding.

^c^ Other category includes any minoritized racial and ethnic group other than Asian, Black, or Hispanic.

One characteristic that differed between the 2 cohorts is the percentage of people with secondary insurance coverage. In months 1 and 18, the 2020 cohorts had a greater percentage of people with secondary coverage compared with the 2018 cohorts, with the difference being larger among adults.

### Changes in Enrollment Over Time

**Figure 1.**
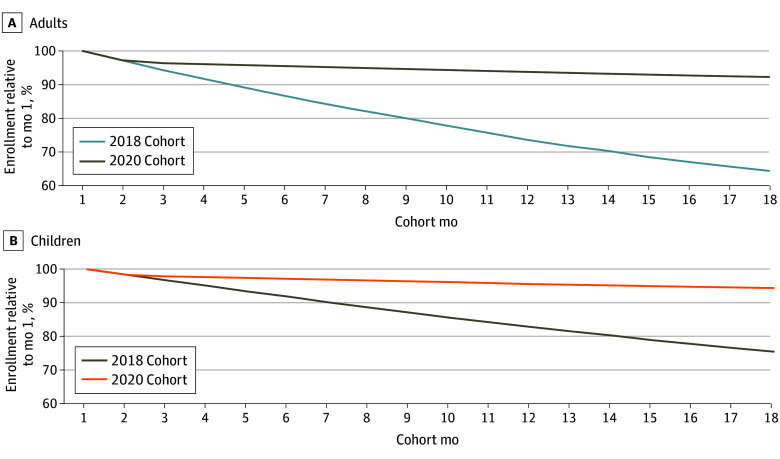
Medicaid Enrollment Compared With Month 1, Prepandemic (2018) and Pandemic (2020) Cohorts Graphs show authors’ analysis of Transformed Medicaid Statistical Information System Analytic Files Research Identifiable Files, 2018 to 2021 for adults (A) and children (B).

At least in part because of the continuous eligibility provision, enrollees in 2020 stayed enrolled for much longer than enrollees before the pandemic (Figure 1). Among adults, the share still enrolled after 18 months was 92.2% (11 065 744 participants) in the 2020 cohort compared with 64.3% (7 942 092 participants) in the 2018 cohort, an increase of 28 percentage points. The share of children still enrolled after 18 months was 94.4% (13 767 655 participants) in the 2020 cohort compared with 75.5% (11 259 064 participants) in the 2018 cohort, an increase of 19 percentage points. Higher persistence in enrollment among children compared with adults in the prepandemic cohort (75.5% vs 64.3%) was consistent with more states having continuous eligibility policies for children prior to the PHE, as well as more generous income eligibility requirements for Medicaid in children.^12^ Despite continuous eligibility coverage for all enrollees in the 2020 cohorts, not all remained enrolled for 18 months because people could still lose Medicaid coverage if they moved states, voluntarily withdrew, or died.

### Changes in Mean Spending Over Time

**Figure 2.**
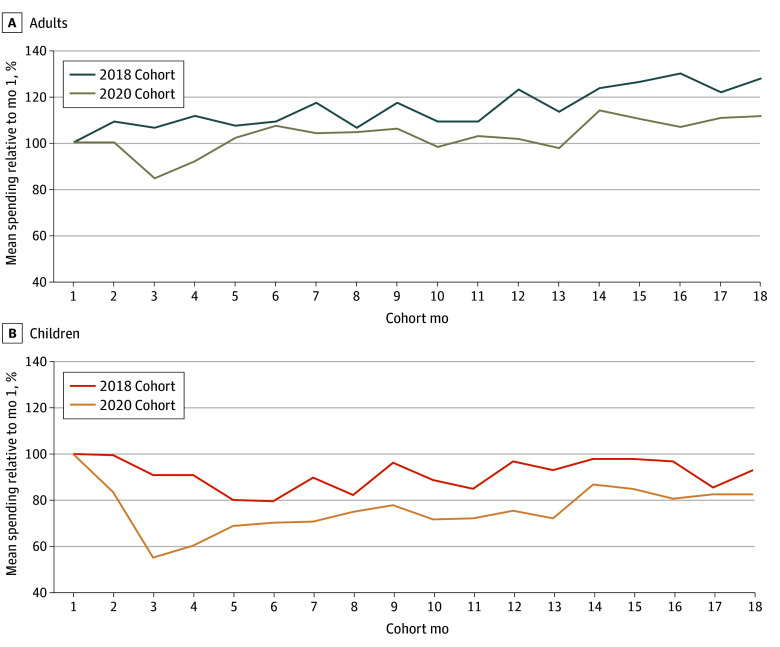
Mean Monthly Medicaid Spending Compared With Month 1, Prepandemic (2018) and Pandemic (2020) Cohorts Graphs show authors’ analysis of Transformed Medicaid Statistical Information System Analytic Files Research Identifiable Files, 2018 to 2021 for adults (A) and children (B).

Compared with the first month, mean spending was generally lower in months 2 to 18 in the 2020 cohorts compared with the 2018 cohorts for adults and children (Figure 2). Some of the observed month-to-month variations in spending were caused by differences in the number of days in the month (see eFigure 2 in Supplement 1). Additional analyses that include adjustments for inflation (not shown) did not substantially change the results of the analyses.

The decline in spending in the 2020 cohorts was pronounced in months 2 through 4, representing March to May of 2020, especially among children. These months corresponded to the height of the pandemic mitigation measures such as lockdowns and social distancing.^13^

For adults, compared with the first month, mean spending in month 3 was 22% lower for the 2020 cohort than for the 2018 cohort (84% vs 106%). Mean spending in month 18 compared with that in month 1 was 17% lower for the 2020 cohort than for the 2018 cohort (111% vs 128%).

Compared with the first month, children’s mean spending in month 3 was 36% lower for the 2020 cohort than for the 2018 cohort (55% vs 91%). Mean spending in month 18, compared with month 1, was 10% lower for the 2020 cohort than for the 2018 cohort (83% vs 93%). Both adults and children in the 2020 cohort had lower spending for all 18 months.

Altogether, we saw evidence of a steep decrease in Medicaid spending per enrollee during the lockdown period, followed by a gradual increase over time. However, even after 18 months, there was still a spending difference between the cohorts, with the pandemic cohorts having lower mean spending on average.

### Additional Analyses

#### Decomposition of Changes in Mean Spending Over Time

**Figure 3.**
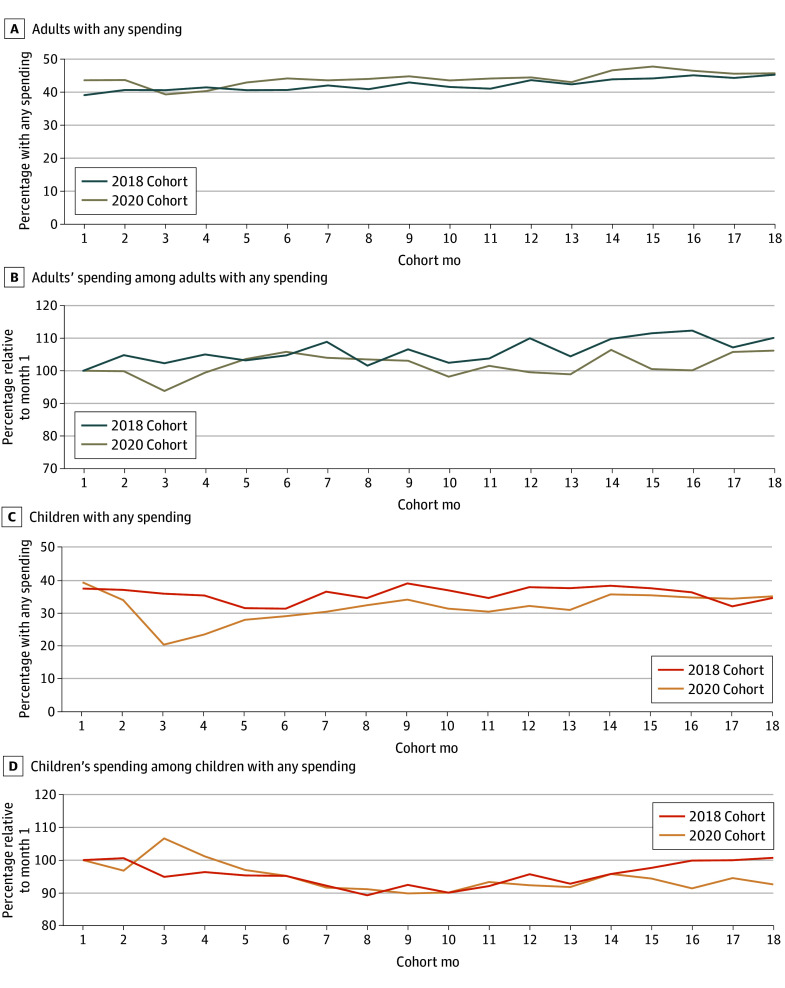
Share With Any Spending and Mean Spending Among Enrollees With Any Spending, Prepandemic (2018) and Pandemic (2020) Cohorts Graphs show authors’ analysis of Transformed Medicaid Statistical Information System Analytic Files Research Identifiable Files, 2018 to 2021 for adults with any spending (A), adults’ spending among adults with any spending (B), children with any spending (C), children’s spending among children with any spending (D).

Our analyses showed that the observed changes in mean spending in 2020 for adults and children were associated with different factors. Although the share of adult enrollees with any spending in the 2020 cohort was slightly higher than the share in the 2018 cohort for most months, the opposite was true for the children’s cohort, which showed a much lower share of enrollees with any spending in 2020 compared with 2018 (Figure 3).

**Figure 4.**
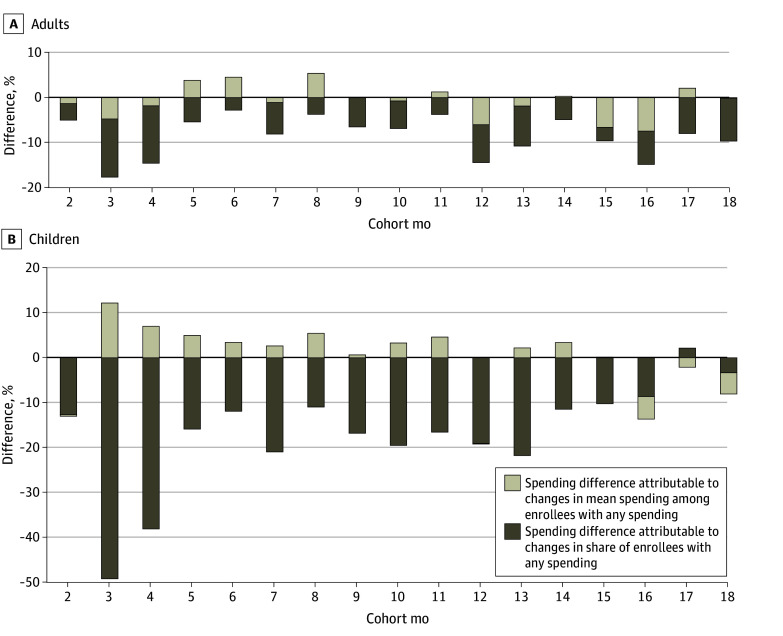
Decomposition of Changes in Mean Monthly Spending Between the Prepandemic (2018) and Pandemic (2020) Cohorts Graphs show authors’ analysis of Transformed Medicaid Statistical Information System Analytic Files Research Identifiable Files, 2018 to 2021, for adults (A) and children (B).

When comparing changes in mean spending among enrollees with any spending across the 2018 and 2020 cohorts, we found that conditional mean spending was higher in some months but lower in other months among adults and higher in almost all months among children. Using this information, Figure 4 shows a decomposition of the observed lower spending in the 2020 cohorts compared with 2018 (see eAppendix 4 in Supplement 1 for a description of the decomposition). For the cohort of adults, we found that the lower mean spending in 2020 could be attributed to both lower mean spending among those with spending and changes in the share of people with any spending in 2020 compared with 2018. The cause of the spending decrease in 2020 was different among the cohort of children. The decrease in the share of children with any spending explained almost all the decrease in monthly mean spending between 2018 and 2020, as shown in Figure 4. In contrast, changes in mean monthly spending among children that spent between the 2018 and 2020 cohorts contributed to higher mean spending in many of the months in the 2020 cohort.

#### Differences in Spending in Month 18 Across the Cohorts

Using a simple calculation, the additional enrollees in month 18 of the 2020 cohort had lower spending compared with those enrolled in month 18 of the 2018 cohort. Our analysis suggests that, in month 18, spending for the additional adults enrolled in 2020 was 58% of spending of adults that would have been enrolled in the absence of the continuous eligibility provision. A similar calculation for children suggested that, in month 18, spending for the additional enrollees in 2020 was 44% of spending among children who would have been enrolled in the absence of the continuous eligibility provision. These calculations divided the share of people enrolled in month 18 of 2020 into the share who would have been enrolled in the absence of the pandemic and continuous eligibility policy as proxied by the 2018 shares (64.3% for adults and 75.5% for children as shown in Figure 1) and the additional share of people enrolled in month 18 of 2020 (27.9% for adults and 18.9% for children). In addition, we assumed that those groups had the same patterns of spending as in 2018, with mean spending in month 18 compared with month 1 being 28% higher among adults and 7% lower among children (Figure 2).

Our calculation suggests that changes in monthly Medicaid spending may have been partially associated with compositional effects. Specifically, people who stayed enrolled in month 18 of 2020 spent less than the people in 2018 as people with lower spending may have lost Medicaid coverage due to lack of a continuous eligibility policy.

## Discussion

As expected, the continuous eligibility provision during the pandemic increased enrollment duration. For both adults and children, this cohort study found lower mean Medicaid spending in the pandemic cohort compared with the prepandemic cohort for the first few months when social distancing and lockdowns were prevalent. For both adults and children, monthly spending rebounded somewhat after the first months of the pandemic, but spending for the pandemic cohort lagged when compared with the prepandemic cohort, even with catch-up in spending over the 18 months. Spending among children lagged by larger percentages for all 18 months.

The observed spending pattern for adults in our analysis is in line with patterns in commercial claims, which showed that spending per person declined from March 2020 to May 2020 but mostly recovered afterward.^14,15,16^ The observed spending pattern for children is also in line with evidence from patterns in spending for ambulatory care services, which suggests that recovery of use of those services was concentrated among the privately insured, with a slower rate of recovery for publicly insured enrollees.^17^

Given little change in the demographic composition of people, the observed sustained changes in mean monthly spending for children could be interpreted in several, mutually exclusive, ways. The cohort of enrollees during the pandemic could be healthier or could have gained access to other sources of coverage. If true, lower mean Medicaid spending is just a reflection of lower need of health care. Instead, if lower mean spending was caused by the pandemic suppressing health care utilization or enrollees being unaware of continuing Medicaid coverage (as suggested by the increase in secondary insurance), the incomplete recovery of spending could have important long-term consequences.

Persistently lower mean spending for children in every month in the pandemic cohort is consistent with other studies reporting that crucial care, such as vaccinations, was deferred.^18,19,20,21^ Exacerbating that outcome, the pandemic was associated with a spike in many other youth health issues, such as poor mental health, lack of school readiness, and sedentary behaviors.^22,23,24^ Another explanation for the lower spending could be lower viral transmission, as children stayed home; weekly influenza surveillance reports and other articles showed a steep decline in the level of influenza and other viruses in children during the lockdown period.^25,26^

For adults, there is some evidence of surgical backlogs, deferred elective operations, and fewer patients seen during the pandemic lockdown period, which could have resulted in lower spending.^27,28,29^ Changes in usage and spending may have also been influenced by the spike in telehealth usage during the first few months of the pandemic.^14,30^

Fruitful avenues for future work could include expanding the length of the analysis with additional data, using all payer claims data to analyze how people switched between Medicaid and private coverage (or became double covered), or looking at rates of utilization of specific medical services such as preventive care and acute care for urgent medical conditions. In addition, further breaking down and examining which types of services (such as outpatient, inpatient, or emergency) drove the reduced spending could better elucidate the mechanisms for spending changes during the PHE.

### Limitations

This study has limitations that should be mentioned. Although the Transformed Medicaid Statistical Information System is the most complete set of Medicaid person-level data and quality has improved greatly, concerns about its reliability remain.^31,32,33^ As a result, many states were omitted. Most importantly, our analysis is descriptive and represents the combined outcomes of the continuous eligibility policy and consequences of the pandemic. In addition, given that we selected February 2020 as their first month for the continuous coverage cohort to make the cohort more comparable to the 2018 cohort, results may not generalize to the broader population affected by continuous coverage, especially individuals who gained coverage after that time.

## Conclusions

In this cohort study, the continuous eligibility provision resulted in individuals being enrolled in Medicaid longer. In addition, mean spending among children and adults was much lower during the pandemic, differences that remained after 18 months. Although the pandemic and continuous eligibility policy increased total Medicaid spending through an increase in the number of people enrolled, these findings suggested that lower mean spending per enrollee moderated that increase. Although moderation of spending may simply reflect lowered utilization of deferrable nonurgent care such in adults, the same deferrable nonurgent care in children may be in the form of preventative care, such as immunizations or well-child visits, which could have implications for long-term health.

## Notes

Supplement 1. **eAppendix 1.** Sample selection: states **eTable 1.** State level analysis of data usability **eFigure 1.** Geographical distribution of states included in analysis **eTable 2.** Characteristics of states included and excluded in analysis **eAppendix 2.** Sample selection: enrollees **eTable 3.** Selection of enrollees included in the analysis **eFigure 2.** Mean monthly Medicaid spending over time by eligibility group, before and during the Pandemic Health Emergency (in nominal dollars) **eAppendix 3.** Comparison of mean spending in month 18 before and during the pandemic **eAppendix 4.** Decomposition calculation

Supplement 2.
Data Sharing Statement

## References

[1] Centers for Medicare & Medicaid Services. January 2023 Medicaid and CHIP enrollment snapshot. Accessed May 5, 2023. https://www.medicaid.gov/medicaid/national-medicaid-chip-program-information/downloads/january-2023-medicaid-chip-enrollment-trend-snapshot.pdf

[2] Frenier C, Nikpay SS, Golberstein E. COVID-19 has increased Medicaid enrollment, but short-term enrollment changes are unrelated to job losses. Health Aff (Millwood). 2020;39(10):1822-1831. doi:10.1377/hlthaff.2020.00900 32757955

[3] Sun R, Staiger B, Chan A, Baker LC, Hernandez-Boussard T. Changes in Medicaid enrollment during the COVID-19 pandemic across 6 states. Medicine (Baltimore). 2022;101(52):e32487. doi:10.1097/MD.0000000000032487 36596028 PMC9803338

[4] Dague L, Badaracco N, DeLeire T, Sydnor J, Tilhou AS, Friedsam D. Trends in Medicaid enrollment and disenrollment during the early phase of the COVID-19 pandemic in Wisconsin. JAMA Health Forum. 2022;3(2):e214752. doi:10.1001/jamahealthforum.2021.4752 35977274 PMC8903121

[5] Khorrami P, Sommers BD. Changes in US Medicaid enrollment during the COVID-19 pandemic. JAMA Netw Open. 2021;4(5):e219463. doi:10.1001/jamanetworkopen.2021.9463 33950210 PMC8100862

[6] Schpero WL, Ndumele CD. Medicaid disenrollment after the COVID-19 pandemic: avoiding a new crisis. JAMA Health Forum. 2022;3(2):e214743. doi:10.1001/jamahealthforum.2021.4743 36218824

[7] Shafer PR, Anderson DM, Whitaker R, Wong CA, Wright B. Association of unemployment with Medicaid enrollment by social vulnerability in North Carolina during COVID-19. Health Aff (Millwood). 2021;40(9):1491-1500. doi:10.1377/hlthaff.2021.00377 34495714

[8] Wright B, Anderson D, Whitaker R, . Comparing health care use and costs among new Medicaid enrollees before and during the COVID-19 pandemic. BMC Health Serv Res. 2021;21(1):1152. doi:10.1186/s12913-021-07027-6 34696801 PMC8544632

[9] McIntyre A, Smith RB, Sommers BD. Survey-reported coverage in 2019-2022 and implications for unwinding Medicaid continuous eligibility. JAMA Health Forum. 2024;5(4):e240430. doi:10.1001/jamahealthforum.2024.0430 38578627 PMC10998158

[10] Sears J, Villas-Boas JM, Villas-Boas SB, Villas-Boas V. Are we #stayinghome to flatten the curve? Am J Health Econ. 2023;9(1):71-95d. doi:10.1086/721705

[11] Blewett LA, Hest R, Lukanen E. Medicaid undercount doubles, likely tied to enrollee misreporting of coverage. State Health Access Data Assistance Center. December 5, 2022. Accessed June 2, 2023. https://www.shadac.org/medicaid-undercount-doubles-likely-tied-enrollee-misreporting-coverage

[12] Conmy AB, Peters C, Lew ND, Sommers BD. Children’s health coverage trends: gains in 2020-2022 reverse previous coverage losses. Assistant Secretary for Planning and Evaluation, Office of Health Policy. March 2, 2023. Accessed November 13, 2023. https://aspe.hhs.gov/sites/default/files/documents/77d7cc41648a371e0b5128f0dec2470e/aspe-childrens-health-coverage.pdf

[13] Farboodi M, Jarosch G, Shimer R. Internal and external effects of social distancing in a pandemic. J Econ Theory. 2021;196:105293. doi:10.1016/j.jet.2021.105293

[14] Sen A, Hargreaves J, Martin K. Effects of COVID-19 on health care spending were concentrated in April-May 2020. Health Care Cost Institute. June 8, 2022. Accessed October 15, 2024. https://healthcostinstitute.org/hcci-originals-dropdown/all-hcci-reports/effects-of-covid-19-on-health-care-spending-were-concentrated-in-april-may-2020

[15] Health Care Cost Institute. 2022 Health care cost and utilization report. April 2024. Accessed October 15, 2024. https://healthcostinstitute.org/images/pdfs/HCCI_2022_Health_Care_Cost_and_Utilization_Report.pdf

[16] Parikh RB, Emanuel EJ, Zhao Y, . Spending patterns among commercially insured individuals during the COVID-19 pandemic. Am J Manag Care. 2023;29(10):517-521. doi:10.37765/ajmc.2023.89440 37870545

[17] Mafi JN, Craff M, Vangala S, . Trends in US ambulatory care patterns during the COVID-19 pandemic, 2019-2021. JAMA. 2022;327(3):237-247. doi:10.1001/jama.2021.24294 35040886 PMC8767442

[18] Fogel B, Schaefer EW, Hicks SD. Early influenza vaccination rates decline in children during the COVID-19 pandemic. Vaccine. 2021;39(31):4291-4295. doi:10.1016/j.vaccine.2021.06.041 34172330 PMC9756823

[19] Chiappini E, Parigi S, Galli L, . Impact that the COVID-19 pandemic on routine childhood vaccinations and challenges ahead: a narrative review. Acta Paediatr. 2021;110(9):2529-2535. doi:10.1111/apa.15949 34028088 PMC8222862

[20] Bramer CA, Kimmins LM, Swanson R, . Decline in child vaccination coverage during the COVID-19 pandemic—Michigan Care Improvement Registry, May 2016–May 2020. MMWR Morb Mortal Wkly Rep. 2020;69(20):630-631. doi:10.15585/mmwr.mm6920e1 32437340

[21] Barach P, Fisher SD, Adams MJ, . Disruption of healthcare: will the COVID pandemic worsen non-COVID outcomes and disease outbreaks? Prog Pediatr Cardiol. 2020;59:101254. doi:10.1016/j.ppedcard.2020.101254 32837144 PMC7274978

[22] Rahman A M, Chandrasekaran B. Estimating the impact of the pandemic on children’s physical health: a scoping review. J Sch Health. 2021;91(11):936-947. doi:10.1111/josh.13079 34494270 PMC8662234

[23] Lebrun-Harris LA, Ghandour RM, Kogan MD, Warren MD. Five-year trends in US children’s health and well-being, 2016-2020. JAMA Pediatr. 2022;176(7):e220056. doi:10.1001/jamapediatrics.2022.0056 35285883 PMC8922203

[24] Mulkey SB, Bearer CF, Molloy EJ. Indirect effects of the COVID-19 pandemic on children relate to the child’s age and experience. Pediatr Res. 2023;94(5):1586-1587. doi:10.1038/s41390-023-02681-4 37280324 PMC10242215

[25] Centers for Disease Control and Prevention. Weekly U.S. influenza surveillance report key updates for week 5, ending February 6, 2021. February 12, 2021. Accessed October 15, 2024. https://stacks.cdc.gov/view/cdc/102734

[26] Yeoh DK, Foley DA, Minney-Smith CA, . Impact of coronavirus disease 2019 public health measures on detections of influenza and respiratory syncytial virus in children during the 2020 Australian winter. Clin Infect Dis. 2021;72(12):2199-2202. doi:10.1093/cid/ciaa1475 32986804 PMC7543326

[27] Mehta A, Awuah WA, Ng JC, . Elective surgeries during and after the COVID-19 pandemic: case burden and physician shortage concerns. Ann Med Surg (Lond). 2022;81:104395. doi:10.1016/j.amsu.2022.104395 35999832 PMC9388274

[28] Schirmer CM, Ringer AJ, Arthur AS, ; Endovascular Research Group (ENRG). Delayed presentation of acute ischemic strokes during the COVID-19 crisis. J Neurointerv Surg. 2020;12(7):639-642. doi:10.1136/neurintsurg-2020-016299 32467244

[29] Aggarwal S, Jain P, Jain A. COVID-19 and cataract surgery backlog in Medicare beneficiaries. J Cataract Refract Surg. 2020;46(11):1530-1533. doi:10.1097/j.jcrs.0000000000000337 32694309 PMC7416873

[30] Mehrotra A, Chernew M, Linetsky D, Hatch H, Cutler D. The impact of the COVID-19 pandemic on outpatient visits: a rebound emerges. The Commonwealth Fund. 2020. Accessed October 15, 2024. https://www.commonwealthfund.org/publications/2020/apr/impact-covid-19-outpatient-visits

[31] US Government Accountability Office. GAO-21-196, Medicaid: data completeness and accuracy have improved, though not all standards have been met. 2021. Accessed December 16, 2022. https://www.gao.gov/assets/gao-21-196.pdf

[32] Saunders H, Chidambaram P. Medicaid administrative data: challenges with race, ethnicity, and other demographic variables. KFF. April 28, 2022. Accessed May 14, 2025. https://www.kff.org/medicaid/issue-brief/medicaid-administrative-data-challenges-with-race-ethnicity-and-other-demographic-variables/

[33] Pervin A, Park C. Update on Transformed Medicaid Statistical Information System (T-MSIS). MACPAC. April 9, 2021. Accessed May 14, 2025. https://www.macpac.gov/wp-content/uploads/2021/04/Update-on-Transformed-Medicaid-Statistical-Information-System-T-MSIS.pdf

