# Supplementary material for: Differences in Medicaid Enrollment and Spending Before and During the COVID-19 Pandemic
Source: JAMA Netw Open. 2025 Jun 18;8(6):e2516569. doi: 10.1001/jamanetworkopen.2025.16569 (PMC12177654; doi:10.1001/jamanetworkopen.2025.16569)
Supplement: Supplement 1. — eAppendix 1. Sample selection: states eTable 1. State level analysis of data usability eFigure 1. Geographical distribution of states included in analysis eTable 2. Characteristics of states included and excluded in analysis eAppendix 2. Sample selection: enrollees eTable 3. Selection of enrollees included in the analysis eFigure 2. Mean monthly Medicaid spending over time by eligibility group, before and during the Pandemic Health Emergency (in nominal dollars) eAppendix 3. Comparison of mean spending in month 18 before and during the pandemic eAppendix 4. Decomposition calculation [file jamanetwopen-e2516569-s001.pdf]

Supplemental Online Content

Hong N, Duchovny N, Ding R. Differences in Medicaid enrollment and spending before and during the COVID-19 pandemic. *JAMA Netw Open*. 2025;8(6):e2516569. doi:10.1001/jamanetworkopen.2025.16569

eAppendix 1. Sample selection: states

eTable 1. State level analysis of data usability

eFigure 1. Geographical distribution of states included in analysis

eTable 2. Characteristics of states included and excluded in analysis

eAppendix 2. Sample selection: enrollees

eTable 3. Selection of enrollees included in the analysis

eFigure 2. Mean monthly Medicaid spending over time by eligibility group, before and during the Pandemic Health Emergency (in nominal dollars)

eAppendix 3. Comparison of mean spending in month 18 before and during the pandemic

eAppendix 4. Decomposition calculation

This supplemental material has been provided by the authors to give readers additional information about their work.

© 2025 Hong N et al. *JAMA Network Open*.

eAppendix 1. Sample selection: states

We limited our sample to 25 states and the District of Columbia with reliable spending and enrollment data as well as constant eligibility rules based on three sources of analysis: CMS's DQ Atlas, which reports data quality of the T-MSIS data for different topics; an analysis from the Department of Health and Human Services; and the authors' analysis of state-level trends. We excluded 1 state due to data quality issues with enrollment and 16 states due to data quality issues with spending (eTable 1).[1] [2] We further omitted 5 states because our own analysis of capitation and payment to providers showed discontinuous or unlikely spending patterns such as significant monthly variation. Last, we omitted 3 states (Missouri, Nebraska, and Oklahoma) that expanded Medicaid during our period of analysis because we expected changes in enrollment and spending in those states that were unrelated to the PHE. The states remaining in our study sample accounted for 53 percent of Medicaid enrollees in 2020.

© 2025 Hong N et al. *JAMA Network Open*.

eTable 1: State level analysis of data usability

State	Data quality concerns based on DQ Atlas		Data concerns based on HHS analysis	Data quality concerns based on authors' analysis		Medicaid expansion
	Enrollment irregularities	Inconsistent total Medicaid spending		Inconsistent capitation spending patterns	Inconsistent MCO payment patterns	
Alabama						
Alaska						
Arizona						
Arkansas		X				
California						
Colorado						
Connecticut						
Delaware						
District of Columbia						
Florida		X				
Georgia						
Hawaii						
Idaho						X
Illinois		X				
Indiana						
Iowa						
Kansas						
Kentucky		X				
Louisiana						
Maine						
Maryland					X	
Massachusetts						
Michigan		X				
Minnesota						
Mississippi			X			
Missouri						X
Montana						

© 2025 Hong N et al. *JAMA Network Open*.

eTable 1: State level analysis of data usability

State	Data quality concerns based on DQ Atlas		Data concerns based on HHS analysis	Data quality concerns based on authors' analysis		Medicaid expansion
	Enrollment irregularities	Inconsistent total Medicaid spending		Inconsistent capitation spending patterns	Inconsistent MCO payment patterns	
Nebraska		X				X
Nevada			X			
New Hampshire						
New Jersey						
New Mexico						
New York		X				
North Carolina						
North Dakota				X		
Ohio						
Oklahoma						X
Oregon		X				
Pennsylvania		X				
Rhode Island	X					
South Carolina						
South Dakota						
Tennessee		X				
Texas						
Utah			X			
Vermont		X				
Virginia						
Washington		X				
West Virginia				X	X	
Wisconsin		X				
Wyoming						

Analysis of Transformed Medicaid Statistical Information System Analytic Files claims data, 2018-2021.

The 25 states we omitted consist of large and small states by both population and area, and there was also no strong geographical basis for which states are omitted (see eFigure 1**)**.

© 2025 Hong N et al. *JAMA Network Open*.

eFigure 1: Geographical distribution of states included in the analysis

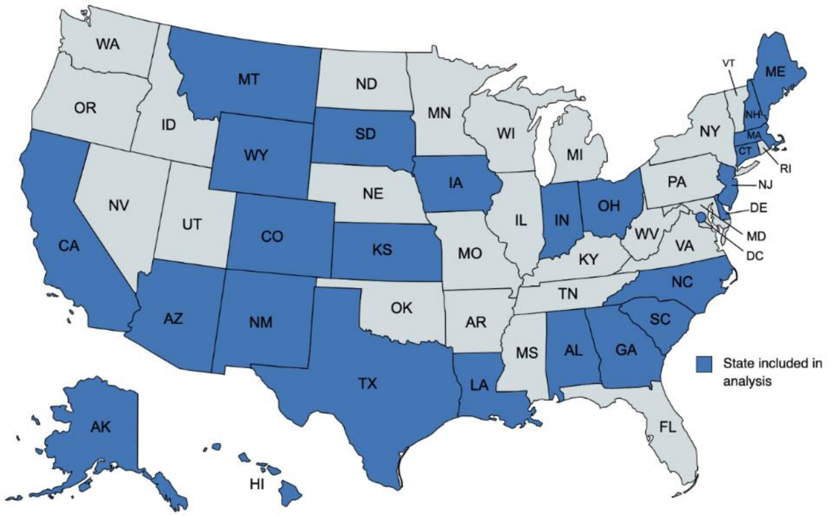

Analysis of Transformed Medicaid Statistical Information System Analytic Files claims data, 2018-2021.

© 2025 Hong N et al. *JAMA Network Open*.

eTable 2. Characteristics of states included and excluded in analysis[a]

	Adults, No. (%)		Children, No. (%)	
	Included States (2018)	Excluded States (2018)	Included States (2018)	Excluded States (2018)
Age, mean (SD), y	37.4 (12.4)	37.4 (12.2)	8.8 (5.2)	8.7 (5.2)
Sex				
Female[b]	7,525,313 (60.9)	6,801,603 (60.5)	7,367,579 (49.4)	6,279,012 (49.5)
Male[b]	4,826,728 (39.1)	4,446,283 (39.5)	7,549,559 (50.6)	6,418,593 (50.5)
Race and ethnicity[b]				
Asian	803,700 (6.5)	552,445 (4.9)	515,886 (3.5)	388,483 (3.1)
Black	1,874,633 (15.2)	1,907,531 (17.0)	2,326,291 (15.6)	2,239,110 (17.6)
Hispanic	3,010,966 (24.4)	1,188,697 (10.6)	4,915,207 (33.0)	2,303,760 (18.1)
White	4,445,842 (36.0)	4,478,718 (39.8)	3,993,395 (26.8)	4,084,869 (32.2)
Other[c]	364,191 (3.0)	156,212 (1.4)	374,673 (2.5)	234,724 (1.9)
Missing Race	1,852,709 (15.0)	2,964,283 (26.4)	2,791,686 (18.7)	3,446,659 (27.1)
Has secondary coverage	1,334,475 (10.8)	1,887,141 (16.8)	1,436,390 (9.6)	1,428,239 (11.2)
Number of individuals	12,352,041	11,247,886	14,917,138	12,697,605

[a]Authors' analysis of Transformed Medicaid Statistical Information System Analytic Files claims data, 2018.

[b]Estimates are rounded to the nearest tenth and might not add up to 100 because of rounding.

[c]Other category includes racial and ethnic minority groups other than Asian, Black, or Hispanic.

© 2025 Hong N et al. *JAMA Network Open*.

eAppendix 2. Sample selection: enrollees

Disabled and aged enrollees were excluded from the analysis because they tend to be enrolled in Medicaid for longer periods than adult and children so that continuous eligibility policies would have a more limited effect among them. We placed several restrictions on the adult and child enrollees included in our analytical samples (eTable 3). We restricted the samples to enrollees with valid age and gender information and full Medicaid benefits (to avoid potential changes in the composition of enrollees that could have been related to the pandemic and could have affected spending). Lastly, we excluded enrollees who died during the follow up period and who had negative monthly spending. To link enrollees across the 18 months of each cohort, we used the state identifier (state_cd) and enrollee identification (msis_id). Individuals in our sample who churned off Medicaid were not added back to the sample for future months if they re-enrolled.

eTable 3: Selection of enrollees included in the analysis

	Adults				Children			
	2018 number	2018 percentage	2020 number	2020 percentage	2018 number	2018 percentage	2020 number	2020 percentage
All individuals enrolled in February	31,464,278	100.0%	30,561,565	100.0%	29,194,393	100.0%	28,283,249	100.0%
Valid age range and gender	27,637,567	87.8%	27,155,454	88.9%	27,839,314	95.4%	26,841,024	94.9%
Full benefit Medicaid enrollees	23,755,345	75.5%	23,508,468	76.9%	27,630,014	94.6%	26,660,331	94.3%
Didn't pass away	23,614,016	75.1%	23,286,866	76.2%	27,619,618	94.6%	26,647,640	94.2%
No negative monthly spending	23,609,574	75.0%	23,284,784	76.2%	27,617,911	94.6%	26,647,025	94.2%
Valid state data based on DQ Atlas and analysis (N=26)	12,352,041	39.3%	11,998,538	39.3%	14,917,138	51.1%	14,585,026	51.6%

Authors' analysis of Transformed Medicaid Statistical Information System Analytic Files claims data, 2018-2021.

© 2025 Hong N et al. *JAMA Network Open*.

eFigure 2: Mean monthly Medicaid spending over time by eligibility group, before and during the Pandemic Health Emergency (in nominal dollars)

A Adults

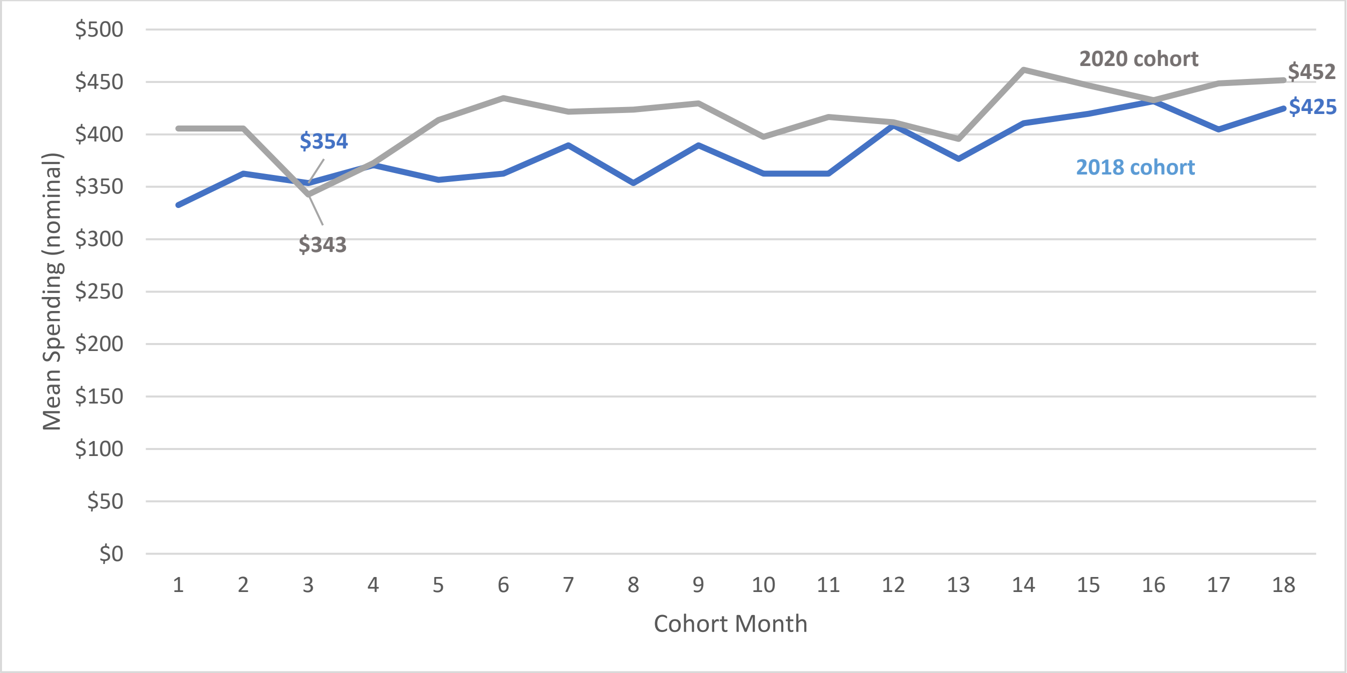

© 2025 Hong N et al. *JAMA Network Open*.

B Children

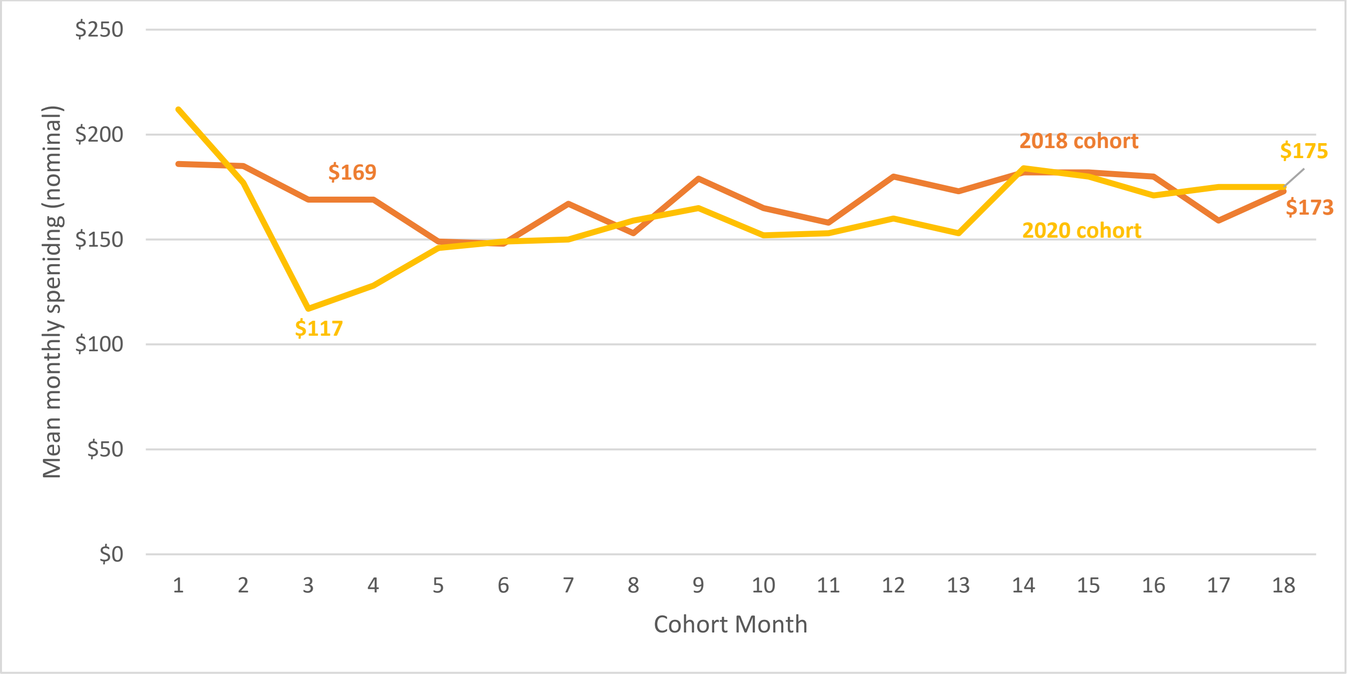

Analysis of Transformed Medicaid Statistical Information System Analytic Files claims data, 2018-2021. Monthly Medicaid spending is calculated as the sum of fee-for-service (FFS) payments and managed care payments to providers.

© 2025 Hong N et al. *JAMA Network Open*.

eAppendix 3. Comparison of mean spending in month 18 before and during the pandemic

We divided enrollees in month 18 of 2020 into two groups: those who would have been enrolled in the absence of the continuous eligibility provision (based on enrollment observed in month 18 of the 2018 cohort) and those who enrolled because of that provision (the difference). Thus, with 92.2% of adults still enrolled in Medicaid in month 18 of 2020 (see Figure 1), we assumed that 64.3% would have been enrolled without the continuous eligibility provision and 27.9% enrolled because of the provision. With 94.4% of children enrolled in Medicaid as of month 18 of the 2020 cohort (see Figure 1), we assumed that 75.5% would have been enrolled without the continuous eligibility provision and 18.9% enrolled because of the provision. Second, we assumed that mean spending in month 18 of the 2020 cohort for the first group of enrollees that would have been enrolled in the absence of the provision was the same as mean spending in month 18 of the 2018 cohort: 128% of month 1 spending for adults and 93% of month 1 spending for children (see Figure 2).

More explicitly, our calculation solves for the weighted average of enrollees at month 18 based on previous spending patterns. We solve for x as the average monthly spending at month 18 for additional enrollees:

2020 Monthly Spending at Month 18 = (2018 Share Enrolled at Month 18/2020 Share Enrolled at Month 18) * 2018 Month Spending at Month 18 + ((2020 Share Enrolled at Month 18 – 2018 Share Enrolled at Month 18)/2020 Share Enrolled at Month 18)*x

For adults, the calculation is as follows: 111% = ((64.3%/92.2%)*127.6%)+((92.2%-64.3%)/92.2%)*x

x = 74%; 74%/128% = 58% of the average adult spending at month 18 for additional enrollees

For children, the calculation is as follows: 83% = ((75.5%/94.4%)*93.0%)+((94.4%-75.5%)/94.4%)*x

x = 41%; 41%/93% = 44% of the average child spending at month 18 for additional enrollees

eAppendix 4. Decomposition calculation

We used the following decomposition to isolate the magnitude of each of changes in any spending and mean spending among enrollees with positive spending:

$$\frac{\mathrm{Si,2020}}{\mathrm{Si,2018}} - 1 \approx \ln(Si, 2020) - \ln(Si, 2018) = \ln\left(\frac{\mathrm{Si,2020}}{\mathrm{Si,2018}}\right) = \ln\left(\frac{\text{Any spendingi,2020}}{\text{Any spendingi,2018}}\right) + \ln\left(\frac{\text{Ave spending among spendersi,2020}}{\text{Ave spending among spendersi,2018}}\right)$$

where $S_{i,2018}$ is the spending in month i relative to spending in month 1 of 2018 cohort, which is equivalent to the product of the probability of any spending in month I relative to spending in month 1 of the 2018 cohort multiplied by the average spending among spenders for that same month i.

[1] Medicaid.gov, DQ Atlas, https://www.medicaid.gov/dq-atlas/welcome.
[2] Department of Health and Human Services. Data on Medicaid managed care payments to providers are incomplete and inaccurate. https://oig.hhs.gov/reports-and-publications/all-reports-and-publications/data-on-medicaid-managed-care-payments-to-providers-are-incomplete-and-inaccurate/.

© 2025 Hong N et al. *JAMA Network Open*.
