# Supplementary material for: Differences in Medicaid Enrollment and Spending Before and During the COVID-19 Pandemic
Source: JAMA Netw Open. 2025 Jun 18;8(6):e2516569. doi: 10.1001/jamanetworkopen.2025.16569 (PMC12177654; doi:10.1001/jamanetworkopen.2025.16569)
Supplement: Supplement 2. — Data Sharing Statement [file jamanetwopen-e2516569-s002.pdf]

Data Sharing Statement

Hong. Differences in Medicaid Enrollment and Spending Before and During the COVID-19 Pandemic. *JAMA Netw Open*. Published June 18, 2025.
doi:10.1001/jamanetworkopen.2025.16569

Data
Data available: No

Additional Information
Explanation for why data not available: Data is proprietary to CMS and cannot be shared
